# Brain Vascular Expression of Monomeric C-Reactive Protein Is Blocked by C10M Following Intraperitoneal Injection in an ApoE-/- Murine Model of Dyslipidemia: An Immunohistochemical Analysis

**DOI:** 10.7759/cureus.60682

**Published:** 2024-05-20

**Authors:** Ylenia Pastorello, Aurelio Pio Russo, Claudia Bănescu, Vittorio Caprio, Zsolt Gáll, Lawrence Potempa, Bogdan Cordoș, Mario Di Napoli, Mark Slevin

**Affiliations:** 1 Department of Anatomy and Embryology, George Emil Palade University of Medicine, Pharmacy, Science and Technology of Târgu Mureș, Târgu Mureș, ROU; 2 Doctoral School of Medicine and Pharmacy, George Emil Palade University of Medicine, Pharmacy, Science and Technology of Târgu Mureș, Târgu Mureș, ROU; 3 Faculty of Medicine in English, George Emil Palade University of Medicine, Pharmacy, Science and Technology of Târgu Mureș, Târgu Mureș, ROU; 4 Center for Advanced Medical and Pharmaceutical Research (CCAMF), George Emil Palade University of Medicine, Pharmacy, Science and Technology of Târgu Mureș, Târgu Mureș, ROU; 5 Department of Genetics, George Emil Palade University of Medicine, Pharmacy, Science and Technology of Târgu Mureș, Târgu Mureș, ROU; 6 Department of Life Sciences, Manchester Metropolitan University, Manchester, GBR; 7 Faculty of Pharmacy, George Emil Palade University of Medicine, Pharmacy, Science and Technology of Târgu Mureș, Târgu Mureș, ROU; 8 Department of Life Sciences, College of Science, Health and Pharmacy, Roosevelt University, Schaumburg, USA; 9 Veterinary Experimental Base, George Emil Palade University of Medicine, Pharmacy, Science and Technology of Târgu Mureș, Târgu Mureș, ROU; 10 Department of Neurological Service, SS. Annunziata Hospital, Sulmona, ITA

**Keywords:** cd105, microvasculature, neurovascular unit, c10m, monomeric c-reactive protein

## Abstract

Introduction

The neurovascular unit (NVU), comprising vascular and glial cells along with neurons, is vital for maintaining the blood-brain barrier (BBB) and cerebral homeostasis. Dysfunction of the NVU is implicated in key neurodegenerative disorders such as Alzheimer's disease (AD). Monomeric C-reactive protein (mCRP), the dissociated form of native, pentameric C-reactive protein (pCRP), is associated with enhanced pro-inflammatory responses in the vascular system, leading to increased permeability and potential NVU disruption.

Methods

This study utilized ApoE-/- mice receiving a high-fat diet which were injected intraperitoneally with either mCRP or mCRP together with a small molecule inhibitor (C10M) and investigated the deposition of mCRP and CD105 expression in the brain parenchyma and its localization within the microvasculature.

Results

Histological analysis revealed significant mCRP deposition in brain microvessels and neurons, indicating potential disruption of the BBB and neuronal damage. Moreover, co-administration of C10M effectively blocked mCRP accumulation in the brain parenchyma, suggesting its potential as a therapeutic agent for effectively inhibiting inflammation-associated degenerative changes. Immunohistochemical staining demonstrated co-localization of mCRP with CD105, indicating potential angiogenic activation and increased susceptibility to inflammatory insult.

Discussion

These findings provide evidence supporting the potential role of mCRP as a contributor to neuroinflammation in individuals with chronic systemic inflammation.

Conclusion

Further studies in human subjects should help validate the efficacy of C10M in preventing or halting neurodegeneration in conditions such as AD and stroke-associated dementia.

## Introduction

Brain, vasculature, neurovascular unit, and neurodegeneration

The functional unit of the brain comprises vascular cells (endothelial cells, pericytes, and vascular smooth muscle cells), glial cells (astrocytes, microglia, and oligodendroglia), and neurons, collectively known as the cerebral neurovascular unit (NVU). Functionality of the NVU is of utmost importance for the integrity of the blood-brain barrier (BBB), and in the modulation of cerebral flow, therefore acting as a calibrator of cerebral homeostasis [1]. Disruption, and consequent dysfunction, of such an intricately regulated system represents the hallmark of neurodeterioration, and pathogenesis of a vast array of neurodegenerative disorders, including Alzheimer's disease (AD), Parkinson's disease, and amyotrophic lateral sclerosis.

Monomeric C-reactive protein

Dissociation of native, pentameric C-reactive protein (pCRP) upon contact with activated cells and tissues, to its monomeric subunits, mCRP, has been associated with a modification of the pro-inflammatory profile of the protein itself. Recruitment of monocytes, activation of macrophage phenotype M1, and upregulation of monocyte chemoattractant protein-1 (MCP-1), and cell adhesion molecules, e.g. intercellular adhesion molecule 1 (ICAM-1), and interleukin 8 (IL-8) in the endothelial cells are some of the mechanisms through which mCRP exerts its noxious sequelae in the vascular system [2]. Specifically, mCRP has been linked to increased vascular monolayer permeability in mouse models. Furthermore, its localization in AD patients' brain microvasculature, as well as the surrounding micro-environment, suggests a role in NVU dysfunction, with possible leakage and perpetuation of damage within the nervous tissue [3,4].

Inhibitors

To date, two small molecules - 1,6-bis(phosphocholine)-hexane (1,6-bis PC) and C10M have been identified as effective dissociation inhibitors [5,6].

1,6-bis PC stabilized pCRP in a decameric conformation and inhibited transformation of pCRP in mCRP in vitro and CRP deposition in inflamed tissues [7,8]. However, this molecule suffers from a short half-life with a rapid clearance, attributed to in vivo hydrolysis of the phosphate moieties of bis-(PC)-H.

To eliminate susceptibility to serum nuclease activity for the compound C10M, the phosphocholine (PC) phosphate moiety was replaced with a phosphonate group. C10M blocked the effects of administered pCRP in rat models, and also monocyte differentiation, and pro-inflammatory M1 phenotypical transition [6,9].

In order to understand the dynamics of mCRP vascular distribution associated with potential neurodegenerative consequences, we investigated the capacity of intraperitoneal (IP) injected mCRP to traverse the BBB, its localization within the parenchyma, and the ability of novel small molecule inhibitors to protect against its accumulation.

## Materials and methods

Production of mCRP

mCRP was characterized and provided by our collaborator Prof. Lawrence Potempa (Roosevelt University, Schaumburg, IL, USA), as described by Slevin et al. [10].

Synthesis of the inhibitor C10M

C10M was synthesized at Manchester Metropolitan University's Department of Life Sciences (UK) using the method reported by Zeller et al., with modifications as given below [6]. A two-step procedure was necessary for preparation.

Diethyl-(3-(dibutylamino)propyl) Phosphonate

At 0 °C, dibutylamine (1.6 g, 12.5 mmol) was added to a DMF (40 mL) solution of diethyl (3-bromopropyl) phosphonate (7.1 g, 27.5 mmol) and sodium iodide (0.15 g, 0.75 mmol). The mixture was agitated at 100 °C for five hours before being cooled to room temperature and acidified with 50 mL of 1M aqueous HCl. The aqueous phase was washed with ethyl acetate (2 × 40 mL), neutralized with solid sodium carbonate, and then extracted with ethyl acetate (2 × 50 mL).

The mixed organic phases were dried and concentrated with anhydrous magnesium sulfate. Column chromatography was used to purify the residue using dichloromethane:methanol (9:1) as the eluent, yielding the title chemical as a colorless oil (1.0 g, 26%).

(3-(Dibutylamino)propyl) Phosphonic Acid (C10M)

Dropwise addition of trimethylsilylbromide (10.0 g, 65.2 mmol) to a solution of diethyl-(3-(dibutylamino)propyl) phosphonate (1.0 g, 3.25 mmol) in dichloromethane (90 mL) at 0 °C was carried out. The liquid was stirred at a low temperature for 12 hours before being cooled to room temperature. Water (150 mL) was added, and the aqueous phase was washed with ethyl acetate (2 × 100 mL) before being concentrated under reduced pressure to get the title compound (0.60 g, 74%). The 1H NMR data was consistent with what had previously been published [6].

Monoclonal antibodies

Mouse monoclonal antibody against human mCRP clone 8C10 was obtained from Dr L.A. Potempa by hybridoma technology and fully characterized, as described previously [10,11]. The non-purified hybridoma culture supernatant was directly used as an experimental 8C10 solution. We have shown its ability to block mCRP, preventing the activation of U937 monocytes [12].

Mouse monoclonal CD105 antibody (OTI8A1, ab156756, Abcam, Cambridge, UK) was used to assess CD105 expression on activated microvascular endothelium and vascular endothelial cells within inflamed brain tissue [13].

Animals and experimental design

Twenty-four ApoE-/-C57BL/6J mice from Jackson Laboratory (Bar Harbor, ME) have been provided with a modified high-fat, Western diet, as detailed by Hintze et al. [14]. Throughout the experiment, the weight and condition of each animal were meticulously monitored. Each mouse was routinely weighed and visually evaluated to determine its overall health. The mice were young adults (12 weeks old) at the start of the study.

After a four-week exclusive high-fat protocol, the 24 mice were categorized into three sets for a further four-week test period: Group 1: receiving a Western diet and IP injections of PBS, as control, (1.1-1.8; n=8), Group 2: Western diet plus IP injections of mCRP (2.1-2.8; n=8), and Group 3: Western diet plus IP injections of mCRP and C10M inhibitor (3.1-3.8; n=8). Mice in groups 2 and 3 received IP injections of 15µg mCRP (average weight 220g), twice per week, for four weeks. Additionally, mice in group 3 received 15µg C10M twice per week, for four weeks, together with mCRP, as aforementioned.

At the end of the test period, the mice were euthanized, and the brains were dissected and fixed in 4% formaldehyde for further histological and immunohistochemical studies.

Staining and immunohistochemistry

The distribution of mCRP was examined using immunohistochemistry (IHC) (mouse anti-human mCRP-specific antibodies 8C10, produced and characterized by Schwedler et al. [15]). After antigen retrieval, an immunohistochemical staining was carried out. Sections were allowed to cool to room temperature after antigen retrieval in 0.01 M sodium citrate buffer, pH 6, heated to 95°C, and then immersed in a 2% hydrogen peroxide solution for 30 minutes. It was then incubated at 4°C for 18 hours with either mouse anti-human mCRP antibody 8C10 or mouse monoclonal CD105 antibody (1:500 in 1% goat serum/0.1% Tween 20/1xPBS).

The next day, antigens were detected in 1% Goat serum/0.1% Tween 20/1xPBS for four hours at RT using a signal stain detection kit, as per the manufacturer instructions (Cell Signaling #36084). Antibody staining was visualized using the HRP/DAB detection system.

No cross-reactivity with the primary antibody was confirmed by inclusion of slides where the secondary antibody was replaced by PBS.

Ethical approval

The animal study protocol was approved by the Ethics Committee of the “George Emil Palade” University of Medicine, Pharmacy, Science and Technology (UMFST) of Targu Mures, protocol code 2158 of 1 March 2023 (“New C-reactive protein inhibitors and dissociation blockers for therapeutic use in cardiovascular diseases-CRE-DICARD”).

## Results

Out of eight brain specimens for each group, four were processed for histology and a representative example is presented.

**Figure 1:**
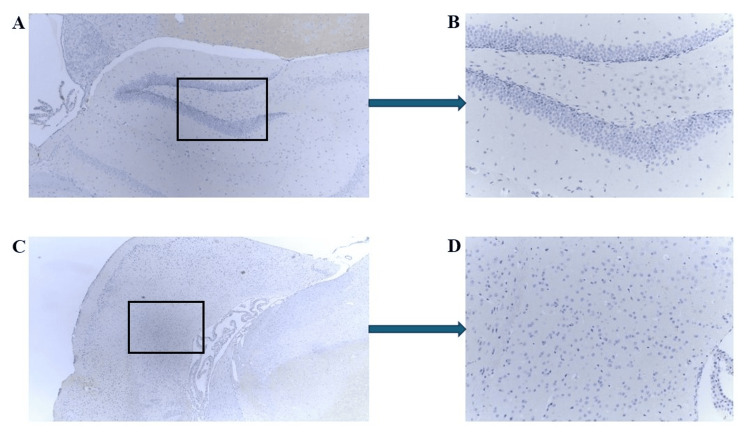
Immunohistochemistry (IHC) of ApoE-/- mouse 1.2 (control group) brain tissue sections following intraperitoneal injections of mCRP. Example staining results from histological and IHC analysis of mouse brain tissue sections (6 μ). (A-B) show the dentate gyrus and hippocampus. No staining for mCRP was seen in any part of the cerebral cortex (A ×40). The boxed area shows a magnified region within the hippocampus (B ×100; boxed area). (C-D) display the cerebral cortex. Staining for mCRP was negative (C ×40). The boxed area shows a magnified region within the cerebral cortex (D ×100; boxed area).

Whilst no mCRP staining was seen in the control untreated mouse brain samples (group 1), all of the four mCRP-treated samples (group 2) showed significant microscopically visible and specifically mCRP-positive regions that were associated primarily with vascular structures and cortical vessels, as well as some neurons within the cerebral cortex. The major highlights were as follows:

No visible expression of mCRP was seen in brain sections of the mice in the control group. The hematoxylin counterstained examples shown in Figure 1 show hippocampal and cortical regions with no observable staining (mouse 1.2; Figures 1A-1B and Figures 1C-1D, respectively).

**Figure 2:**
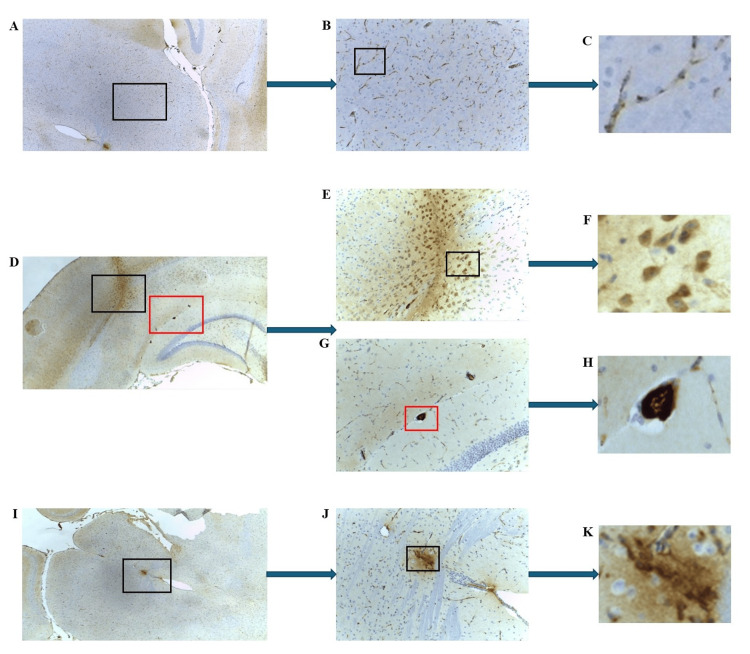
IHC of ApoE-/- mouse 2.1 (mCRP-injected group) brain tissue sections following intraperitoneal injections of mCRP. Example staining results from histological and IHC analysis of mouse brain tissue sections (6 μ). (A-C) highlight strong mCRP-positivity in microvessels within the midbrain (A ×40; B ×100 and C ×400; boxed areas). (D-H) show notable mCRP staining within a cluster of specific primary somatosensory cortex neurons (D ×40; black box E ×100, F ×400) and in microvessels and moderately-sized vessels within and around the corpus callosum (red box G ×100, H ×400). (I-K) display pronounced mCRP-positivity in neurons and microvessels of the midbrain (I ×40). The magnified box shows a cluster of positively stained neurons (J ×100 and K ×400). IHC: Immunohistochemistry

Notable mCRP staining was identified in the midbrain region, specifically, in many of the visible microvessels (mouse 2.1; Figures 2A-2C), and in mesencephalic neurons, as evidenced by the cluster shown in Figures 2I-2K. In addition, both microvessels and moderately sized vessels within and around the corpus callosum (mouse 2.1; Figures 2D, 2G, 2H, red box) stained positively for mCRP. Furthermore, localization of mCRP was seen within a cluster of specific primary somatosensory cortex neurons, as shown in Figures 2D-2F (black box).

**Figure 3:**
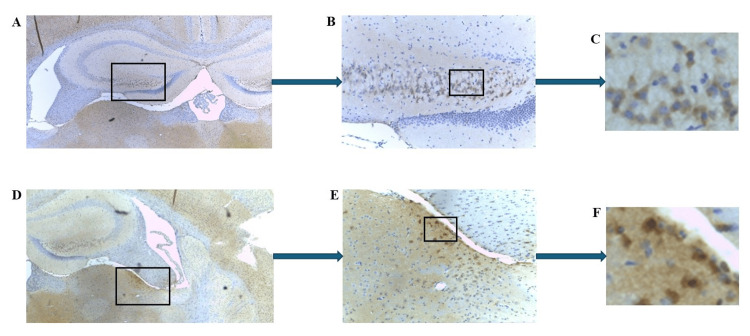
IHC of ApoE-/- mouse 2.3 (mCRP-injected group) brain tissue sections following intraperitoneal injections of mCRP. Example staining results from histological and IHC analysis of mouse brain tissue sections (6 μ). (A-C) demonstrate high mCRP-positivity in hippocampal neurons (A ×40; B ×100 and C ×400; boxed areas). (D-F) show strong mCRP-positivity in neurons of the metathalamus (D ×40). Positive neurons are highlighted in the boxes (E ×100 and F ×400). IHC: Immunohistochemistry

In mouse 2.3, several areas displayed positive staining for mCRP. Figures 3A-3C show perinuclear and cytoplasmic expression within the hippocampal region, whereas Figures 3D-3F present deposition within the lateral geniculate body of the metathalamus.

**Figure 4:**
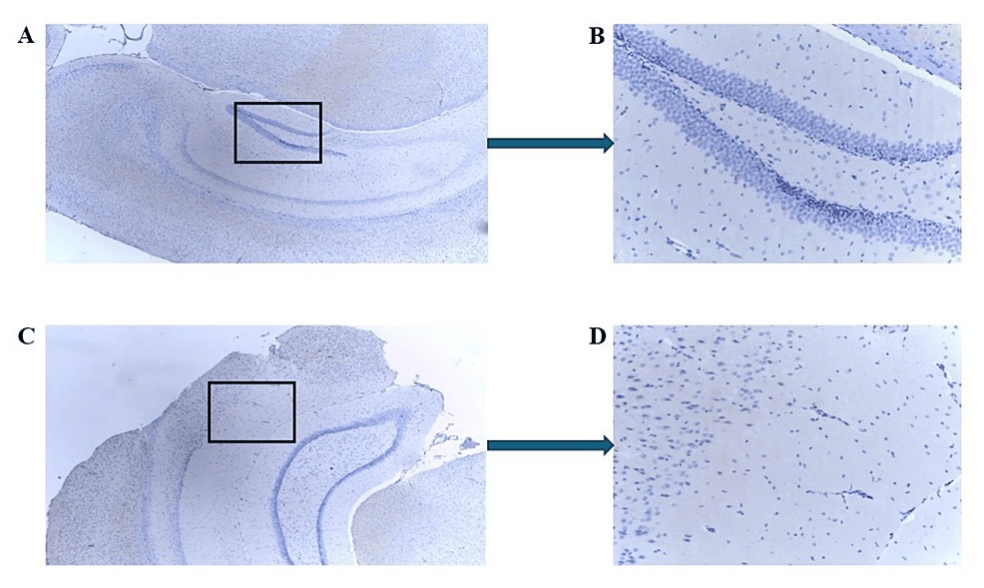
IHC of ApoE-/- mouse 3.2 (mCRP plus C10M-injected group) brain tissue sections following intraperitoneal injections of mCRP. Example staining results from histological and IHC analysis of mouse brain tissue sections (6 μ). (A-B) show the hippocampal formation. No positive mCRP staining was detected (A ×40; B ×100; boxed area). (C-D) display the cerebral cortex. Staining for mCRP was negative (C ×40; D ×100; boxed area). IHC: Immunohistochemistry

When the mCRP inhibitor C10M was co-injected with mCRP, its deposition appeared to be completely blocked. Figure 4 shows the mouse 3.2 negatively stained hippocampal region and cerebral cortex (Figures 4A-4B and Figures 4C-4D, respectively).

**Figure 5:**
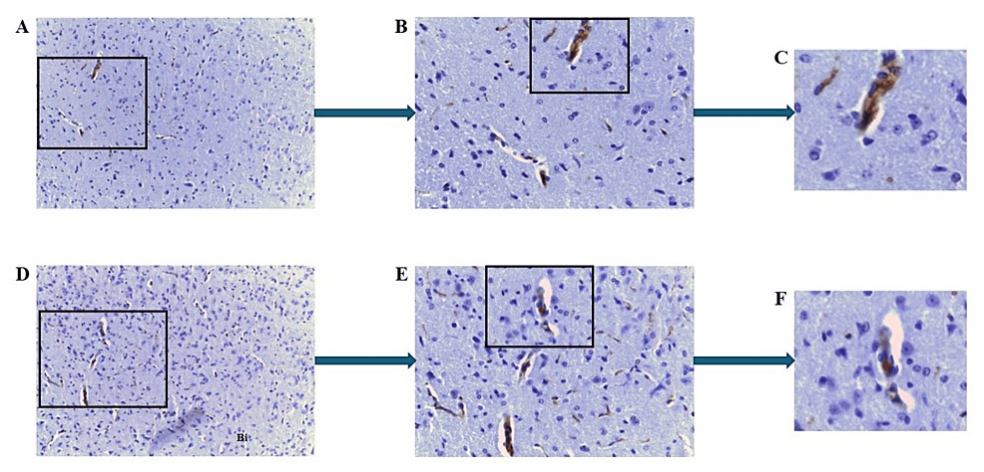
IHC of ApoE-/- mouse 2.1 (mCRP-injected group) showing multiple labeling for CD105/mCRP within the cortical microvessels, in serial sections. Example staining results from histological and IHC analysis of mouse brain tissue sections (6 μ). (A-C) highlight strong mCRP-positivity in the cortical microvessels (A ×100; B ×200 and C ×400; boxed areas). (D-F) demonstrate a serial section stained with CD105 antibody, showing positive cortical microvessels in the same location as the mCRP staining in A-C (D ×100; E ×200 and F ×400; boxed areas). IHC: Immunohistochemistry

Serial sections from mouse 2.1 were subjected to staining with anti-mCRP antibody or anti-CD105 antibody in order to ascertain potential co-localization of these proteins in the brain. Figures 5A-5C show positive mCRP staining within cortical microvessels, whilst Figures 5D-5F show the corresponding serial section and specifically highlighted microvessels that were concomitantly stained positively for CD105.

**Figure 6:**
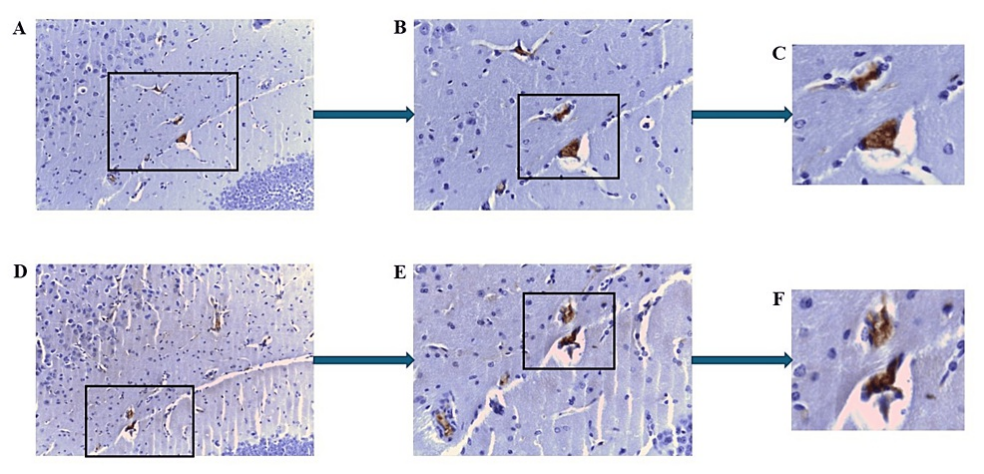
IHC of ApoE-/- mouse 2.1 (mCRP-injected group) brain tissue showing multiple labeling for CD105/mCRP within moderately sized blood vessels of the corpus callosum, in serial sections. Example staining results from histological and IHC analysis of mouse brain tissue sections (6 μ). (A-C) display notable mCRP-positivity in vessels of the corpus callosum (A ×100; B ×200 and C ×400; boxed areas). (D-F) demonstrate a serial section stained with CD105 antibody showing positive vessels in the same location as the mCRP staining in A-C (D ×100; E ×200 and F ×400; boxed areas). IHC: Immunohistochemistry

Figures 6A-6C display observable mCRP expression within moderately sized blood vessels of the corpus callosum, whilst Figures 6D-6F confirm co-localization in the same vessels with CD105.

**Figure 7:**
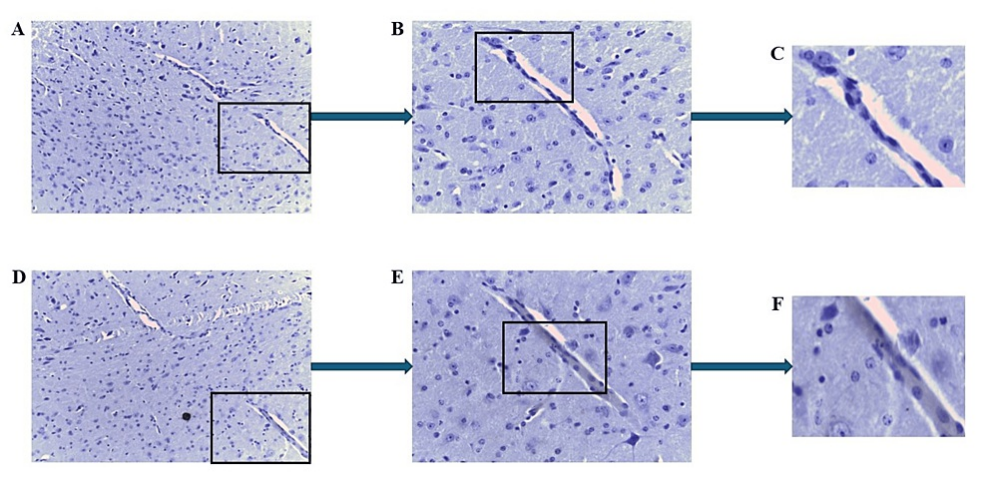
IHC of ApoE-/- mouse 3.2 (mCRP plus C10M-injected group) brain tissue showing negative staining for CD105/mCRP within cortical blood vessels, in serial sections. Example staining results from histological and IHC analysis of mouse brain tissue sections (6 μ). (A-C) highlight cortical tissue, with the sample being negative for mCRP (A ×100; B ×200 and C ×400; boxed areas). (D-F) show a serial section in the same location as A-C, negative for CD105 staining (D ×100; E ×200 and F ×400; boxed areas). IHC: Immunohistochemistry

In contrast, when mCRP was injected with C10M, neither mCRP nor CD105 was observed in any of the brain tissue samples, as shown in Figure 7 (Figures 7A-7C: mCRP; Figures 7D-7F: CD105), which were negatively stained for both (black box showing the negatively stained blood vessels).

## Discussion

The murine ApoE-/- knock-out models represent a useful proof-of-concept system with features similar to those seen in human neurodegenerative processes. Specifically, the ApoE proteins are responsible for lipid transport and cholesterol homeostasis within the central nervous system, and ApoE knock-out is associated with Tau and Amyloid beta (Aβ) aggregation, microglia activation, BBB disruption, and accelerated symptoms of dementia [16].

Here we showed that multiple direct IP injections of mCRP over a period of four weeks into young ApoE-/- mice, concomitant with a high-fat diet, resulted in significant transfer of the protein into the brain parenchyma. A recent study by Zhang et al. showed that middle-aged ApoE knock-out mice, injected over a period of six weeks (three times per week IP with mCRP), resulted in co-localization of mCRP with endothelial cells (CD31 binding) but not neurons or astroglia in the brain, with concomitant reduction in cerebrovascular length. mCRP also increased the number of CD3/8 T-lymphocytes within the brain and the expression of p-Tau, a specific marker of neurodegeneration [17]. In another study, Na et al. (2023) showed that elevation of plasma mCRP was associated with reduced ApoE expression in mice, together with a reduction in pericytes, concomitant with brain tauopathy and increased neuroinflammation [18].

In our study, we focused on the identification of deposition of mCRP within specific brain regions and its ability to activate the microvasculature. Animals receiving 70µg/Kg twice a week for four weeks of mCRP demonstrated notable expression in widespread regions, namely hippocampus, cerebral cortex, corpus callosum, mesencephalon, and metathalamus. Staining was seen within microvessels (in the vessel walls and lumen), and neurons (mostly perinuclear staining) of these areas. IP injection of substances infers dissemination via the mesenteric vessels, portal system, and hepatic metabolism before reaching the systemic circulation; hence, it is likely that mCRP transfers to the brain microvasculature via this mechanism following systemic induction of inflammation, increasing the permeability of the BBB [19]. Within the brain parenchyma, deposited mCRP within the microvessels, could lead to disruption of the endothelial cell junctions, as was shown previously, which could explain leakage into the extracellular matrix and subsequent uptake by local neurons [3]. Similar to the findings by Gan et al. and Garcia-Lara et al., we did not find any notable expression of mCRP in cells with the morphology of glia or microglia; however, the expression by neurons could lead to cytotoxicity as demonstrated by an increase in TUNEL and cleaved caspase-3 expression in addition to tau hyperphosphorylation [20,21].

Previous work showed that mCRP was deposited in the cortical microvessels of individuals who died from either stroke or AD, and this was concomitant with expression of inflammatory markers, such as CD68 and interleukin-1 beta (IL-1β), and also aquaporin 4, associated with lymphatic Aβ clearance [4,22]. Here, we showed a strong co-localization of mCRP with endoglin (CD105) in the lumen and in the intimal endothelial cells of small to medium-size cortical microvessels in serial sections of mice who were injected with mCRP but not in control, untreated mice. This suggests potential angiogenic activation of the vessels, supporting their increased permeability and susceptibility to inflammatory insult, leakage, and degeneration [23,24].

In this study, we investigated the potential of a novel small molecule biological inhibitor (C10M; designed originally to block pCRP dissociation to the monomer) to prevent the deposition of mCRP within the brain, following its IP co-injection in a test group of mice. The pro-inflammatory activity of mCRP follows CRP dissociation on cell membrane phosphocholine residues via the action of phospholipase A2, and several inhibitors based upon abrogation of this process have been recently described [6,25]. Notably, intravenous (i.v.) administration of the C10M inhibitor blocked mCRP-induced ischemia/reperfusion injuries within the renal tissue of rat models, and mCRP localization was not detected. C10M administration was furthermore associated with improved excretory renal function [6]. When 1,6-bis PC was co-infused i.v. with pCRP in a rat model of myocardial ischemia/reperfusion, the formation of mCRP within the infarcted tissue was completely abolished [7].

We found that unlike the mCRP-treated animals alone, where the expression/deposition of the protein was widespread throughout the brain, those animals who received the C10M inhibitor concurrently showed a complete lack of mCRP deposition within the brain.

In terms of the potential mechanism through which this occurred, since we recently observed that C10M abrogated mCRP-induced clustering, macrophage M1 activation, and pro-inflammatory cytokine secretion, it is possible that direct binding of this small molecule inhibitor to mCRP could reduce its ability to induce systemic inflammation necessary for BBB disruption in our experimental model and, hence, blocking its entrance into the brain parenchyma [9].

Limitations of the study

Future work should implement blood biomarker analysis from the examined mice, as well as additional use of markers of inflammation in the IHC, in order to provide a more comprehensive understanding of the dynamics involved in the investigated processes.

## Conclusions

mCRP may contribute to NVU dysfunction by its deposition within the brain microvessels, disruption of the endothelial cell architecture, and subsequent spreading in the surrounding cerebral neurons. Prevention of this pathological process could be achieved by administration of the novel small molecular inhibitor C10M which, in this study, demonstrated efficacy in completely abolishing accumulation of mCRP and expression of CD105 within the mice brain parenchyma and microvasculature. Further studies involving human subjects are needed in order to assess the potential of C10M in prevention (e.g. dementia after stroke) or blocking of neurodeterioration in early diagnosed AD patients.
